# Utilization of multiple genetic methods for prenatal diagnosis of rare thalassemia variants

**DOI:** 10.3389/fgene.2023.1208102

**Published:** 2023-07-17

**Authors:** Fan Jiang, Jianying Zhou, Liandong Zuo, Xuewei Tang, Jian Li, Fatao Li, Tianhe Yang, Yanxia Qu, Junhui Wan, Can Liao, Dongzhi Li

**Affiliations:** ^1^ Prenatal Diagnostic Center, Guangzhou Women and Children’s Medical Center Affiliated with Guangzhou Medical University, Guangzhou, Guangdong, China; ^2^ Xiangya School of Nursing, Central South University, Changsha, Hunan, China

**Keywords:** rare thalassemia, prenatal diagnosis, SMRT sequencing, next-generation sequencing, complex genotypes

## Abstract

**Background:** Thalassemia is the most prevalent monogenic disorder caused by an imbalance between the α- and β-globin chains as a result of pathogenic variants in the α- or β-globin genes. Novel or complex structural changes in globin genes are major hurdles for genetic consulting and prenatal diagnosis.

**Methods:** From 2020 to 2022, genetic analysis was performed on 1,316 families suspected of having children with thalassemia major, including 42 pregnant couples suspected of being thalassemia carriers with rare variants. Multiple techniques including multiplex ligation-dependent probe amplification (MLPA), Sanger sequencing, targeted next-generation sequencing, and single-molecule real-time (SMRT) sequencing were used to diagnose rare thalassemia.

**Results:** The rate of prenatal diagnosis for rare thalassemia variants was 3.19% (42/1,316). The most prevalent alleles of α- and β-thalassemia are Chinese ^G^γ(^A^γδβ)^0^and -- ^THAI^ deletion. In addition, ten rare complex genotypes include one Chinese ^G^γ(^A^γδβ)^0^ deletion combined with *HBG1-HBG2* fusion, two rare deletions at HBB gene (hg38, Chr11: 5224211-5232470, hg38, Chr11: 5224303-5227790), one complete 7,412 bp fusion gene for anti-Lepore Hong Kong, two complex rearrangements of the α-globin gene cluster, two novel duplications, and two rare large deletions in the α-globin gene cluster.

**Conclusion:** Accurate gene diagnosis for probands with combined molecular biology techniques is the key to prenatal diagnosis of rare thalassemia.

## 1 Introduction

Thalassemia, primarily including α- and β-thalassemia, is one of the most prevalent birth abnormalities in south China, (Lai et al., 2017). The imbalance between α- and β-globin chains determines the severity of clinical symptoms. Thalassemia’s clinical symptoms can vary from asymptomatic hemolytic anemia to fatal hemolytic anemia (Kattamis et al., 2022). The identification of high-risk partners through thalassemia carrier screening is an effective prevention of the birth of neonates with severe thalassemia. Hematological and biochemical tests are generally used first to identify individuals with phenotypic characteristics, followed by disease identification using genetic testing. Gap-polymerase chain reaction (gap-PCR), reverse dot-blot assay (RDB), and real-time PCR-based multicolor melting curve analysis (MMCA assay) are frequently used in China to detect common thalassemia genotypes, including four common α-thalassemia deletions, three common non-deletional α-thalassemia mutations, and seventeen known β-thalassemia mutations (Jiang et al., 2021a). However, approximately 2% of mutations cannot be detected using these techniques, making prenatal diagnosis of rare types of thalassemia mutations difficult (Li, 2017).

Molecular biological techniques have been developed rapidly in recent years, which significantly improved the diagnosis of rare thalassemia genotyping. Multiplex-ligation dependent probe amplification (MLPA) and Sanger sequencing are popular methods commonly used for detecting rare copy-number variations (CNV) and mutations. However, there are limitations associated with both methods. For example, Sanger sequencing produces high-quality sequences of only up to 1 kb (Krier et al., 2016). MLPA only identifies changes within the probes’ target sequence. Copy number neutral inversions or translocations remain undetected using both methods, making inversions or translocations with copy number neutrality unnoticed.

Due to high homology and high GC-rich regions of the *HBA2* and *HBA1* genes, the accuracy of NGS (next-generation sequencing) methods based on short reads is not optimal for globin cluster variants (Xu L. et al., 2020). Third-generation sequencing (TGS) has been used in thalassemia genetic testing recently. This method is based on Pacific Bioscience (PacBio) single-molecule real-time sequencing (SMRT) and has been proven to be able to detect structure variations (SVs), especially the ability to effectively and accurately distinguish complex rearrangement due to its advantages of long reads and single-molecule resolution. In clinical settings, to reduce errors in raw subhead coverage and decrease costs, SMRT is commonly performed following multiplex long-molecule PCR. However, it remains challenging to provide more precise information on positions for unknown deletions or duplications that occur outside of the detection range. In this study, we conducted a thorough analysis of thalassemia alleles for a better prenatal diagnosis and counseling of thalassemia carriers.

## 2 Materials and methods

### 2.1 Samples and hemoglobin testing

A total of 1316 pregnant women and their husbands who attended the Guangzhou Women and Children Medical Center from January 2020 to December 2022 were included in this study. The 1,316 subjects include 805 pregnant women who underwent chorionic villus sampling (12.4 weeks of average pregnancy) and 511 pregnant women who underwent amniocentesis (18.5 weeks of average pregnancy, 15 twin pregnancy cases included). All samples in this study were obtained with the approval of the Guangzhou Women and Children Medical Center’s Ethics Committee. Participants signed the informed consent after a full explanation. Couples carrying common variants were clarified first by receiving conventional molecular analysis before prenatal diagnosis. Couples suspected to be carriers with rare genotypes first received routine hematology examination and Hb electrophoresis and then performed other genetic tests for determining thalassemia genotypes. Prenatal diagnosis was performed in families at risk of having affected offspring after a definite diagnosis. Blood samples collected using the K2EDTA tubes were used for routine blood analysis, and automatic high-pressure liquid-flow capillary electrophoresis was used for hemoglobin analysis to detect HbF, HbA2, HbH, and other hemoglobin variants (CE; Serbia, Paris, France). Normal ranges were MCV ≥80 fL, MCH ≥27 pg, 2.5% < Hb A2 < 3.5%, and Hb F < 5.0%.

### 2.2 Conventional molecular analysis

Genomic DNA was isolated from whole blood leukocytes using the DNA Isolation Kit (Zhishan Inc., Xiamen, China) according to the manufacturer’s protocol. The MeltPro *HBB* and *HBA* assays (Xiamen Zeesan Biotech Co., Ltd., Xiamen, Fujian, China) were used to simultaneously detect common genotypes including four common deletional α thalassemia (--SEA, --THAI, –α3.7 and–α4.2), three nondeletional α thalassemia (*HBA2*: c.427T>C, *HBA2*: c.369C>G, *HBA2*: c.377T>C), and 21 single-nucleotide mutations in the HBB gene (*HBB*: c.-140C>T, *HBB*: c.-123A>T, *HBB*: c.-78A>G, *HBB*: c.-79A>G, *HBB*: c.-80T>C, *HBB*: c.-81A>C, *HBB*: c.-82C>A, *HBB*: c.45_46insG, *HBB*: c.48_49insG, *HBB*: c.52A>T, *HBB*: c.79G>A, *HBB*: c.91A>G, *HBB*: c.92+1G>T, *HBB*: c.92+5G>C, *HBB:* c.84_85insC, *HBB*: c.113G>A, *HBB*: c.216_217insA, *HBB*: c.130G>T, *HBB*: c.124_127delTTCT, *HBB*: c.315+5G>C, *HBB*: c.316-197C>T). 20 uL of PCR mix and 5 uL of extracted genomic DNA were used for this assay on the SLAN-96P real-time system (Hongshi Medical Technology Co., Ltd., Shanghai, China).

### 2.3 Molecular analysis for rare thalassemia variants

Sanger sequencing of the entire α-globin genes (α1 and α2) and β-globin gene was carried out to identify unidentified mutations. MLPA (MRC-Holland, Amsterdam, Netherlands) was utilized to detect deletions and duplications within α and β-globin genes. Gap-PCR and sequence analysis using special flanking primers were further used to indicate the breakpoints in those samples.

DNA samples suspected to be rare complex thalassemia were purified and quantified using the Qubit dsDNA BR assay kit (Thermo Fisher Scientific, Waltham, MA, United States). Single-molecule real-time (SMRT) sequencing by Pacific Biosciences was performed after optimized multiplex long PCR for detecting SNVs and indels in *HBA1, HBA2,* and *HBB* genes. Dumbbell-shaped PacBio pre-libraries were prepared and purified, then SMRT bell libraries were generated using the Sequel Binding and Internal Ctrl Kit 3.0 (Pacific Biosciences). Primed DNA-polymerase complexes were loaded onto SMRT cells (Pacific Biosciences) and sequenced on the PacBio Sequel II System. CCS software (Pacific Biosciences) was used for analyzing and CCS reads were aligned to genome build hg38 by pbmn2. Single nucleotide variations (SNVs), small insertions and deletions (indels), and structure variations (SVs) were distinguished using FreeBayes (https://www.geneious.com/plugins/freebayes; Biomatters, Inc., San Diego, CA). SNP-based haplotype analysis (cis or trans) was performed using the WhatsHap (version 0.18) software and pb ampliconclustering software. Samples with a large duplication in the α-globin gene cluster were sent to BGI Genomics for targeted next-generation sequencing. The total size of the targeted sequences was ∼275,234 bp including the protein-coding regions, key regulatory regions, known pathogenic copy number variants (CNVs) regions, Single nucleotide variations (SNVs)/insertion and deletion variants (indels) in the non-coding regions of hemoglobin gene clusters (α- and β-globin gene clusters), and four modifier genes (KLF1, BCL11A, HBS1L, and MYB). After library preparation and sequencing on HiSeq2000 or 4000 sequencers, the raw fastq data was automatically delivered to an in-house integrated pipeline (Shang et al., 2017). Pathogenicity of variants or CNV was classified based on the guidelines of the American College of Medical Genetics (ACMG) and literature (Richards et al., 2015).

### 2.4 Short tandem repeat (STR) analysis

All the prenatal samples were identified for maternal cell contamination using the Human Trisome (T21, T13, T18, and Sex) Polyploidy Analysis kit (DAAN Gene Co., Ltd. of Sun Yat-sen University; Guangzhou, China), which included 4 STR foci on chromosome 13 (D13S628, D13S742, D13S634, and D13S305), 4 foci on chromosome 18 (D18S1002, D18S391, D18S535, and D18S386), 3 foci on chromosome 21 (D21S1435, D21S1411, and D21S11), and 3 foci on a sex chromosome (DXS981, DXS6809, and X22).

## 3 Results

### 3.1 Results for prenatal diagnosis of rare thalassemia

**FIGURE 1:**
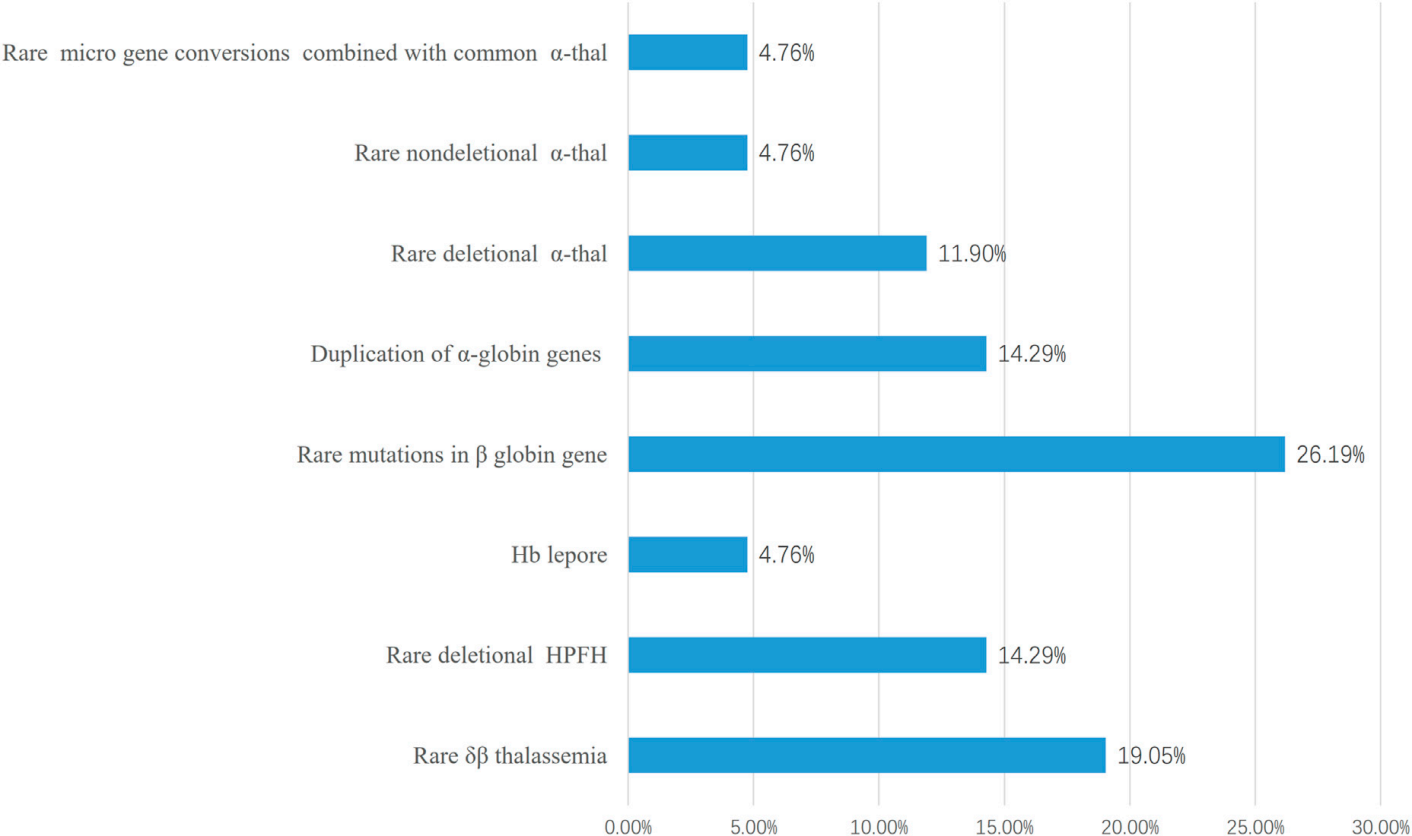
The main cause of prenatal diagnosis in those 42 families at high risk of giving birth to babies of transfusion-dependent Anemia. 30 families giving birth to babies of severe β-thalassemia and 12 families giving birth to babies with Hb Bart’s hydrops fetalis or Hb H disease were included. The most common cause was rare deletional β-thal including δβ thalassemia (8/42) and deletional HPFH(6/42).

In the 1,316 families, the common genetic diagnosis of 1,274 couples was consistent with previous results. The 42 probands from the remaining families reached an accurate diagnosis using different molecular methods. All women enrolled in the study underwent prenatal diagnosis. The most common genotype in those 42 at-risk couples was Chinese ^G^γ(^A^γδβ)^0^ (8/42, 19.05%), followed by SEA-HPFH (4/42, 9.52%). The--THAI deletion is most common in families who were at high risk of having children with rare severe α thalassemia (3/42, 7.14%). The -α^11.1kb^ (chr11:5224211-5232470) first reported by our team was another common genotype of rare α thalassemia in those 42 families (2/42, 4.76%). Besides rare deletion and rare mutation in the *HBB* gene, duplication of α-globin genes coexisted with β thalassemia was also a common cause for prenatal diagnosis to avoid children with rare beta thalassemia-major (6/42, 14.28%). The spectrum of rare genotypes was described in Table 1 and the main cause of prenatal diagnosis in those 42 families was shown in Figure 1. STR analysis of prenatal samples showed no maternal cell contamination. The prenatal diagnosis rate for rare types of thalassemia was about 3.04%. Prenatal diagnosis showed 330 affected fetuses, including 97 with severe β-thal syndrome, 202 with Hb Bart’s hydrops fetalis, and 31 with nondeletional HbH. Ten cases with complex or novel α/β globin gene cluster structural variants were assessed. Clinical and molecular information was described in Table 2.

**TABLE 1:** Rare genotypes and Frequency of α/β thalassemia and detected methods in 42 families.

Common name	HGVS name	*N* (%)
Chinese ^G^γ(^A^γδβ)^0^	NC_0000011.9:g.5191148_5270051del	8 (22.22)
SEA-HPFH	NC_0000011.9:g.5222878_5250288 del	4 (11.11)
CD37 (TGG>TAG)	HBB:c.113G>A	2 (5.56)
−90 (C>T)	HBB:c.-140C>T	1 (2.78)
−88 (C->T)	HBB:c.-138C>T	1 (2.78)
−72 T>A	HBB:c.-122T>A	1 (2.78)
HBB:c.^*^32A>C	HBB:c.^*^32A>C	1 (2.78)
Codons 8/9 (+G)	HBB:c.27_28insG	1 (2.78)
Hb G-Siriraj	HBB:c.22G>A	1 (2.78)
HBB:c.383A>G	HBB:c.383A>G	1 (2.78)
	NC_0000011.9:g. 5245532_5249019 del	1 (2.78)
	NC_0000011.9:g. 5245440_5253700 del	1 (2.78)
	NC_0000011.9:g. 5275498_5280421 del	1 (2.78)
Hb Dieppe	HBB:c.383A>G	1 (2.78)
Hb Lepore Hong Kong	NC_0000011.9:g. 5248181_5255592 del	1 (2.78)
Hb anti-Lepore Hong Kong	NC_0000011.9:g. 5248181_5255592 dup	1 (2.78)
Hb Heze	HBB:c.434A>G	1 (2.78)
--THAI	NC_0000016.9:g. 199800_233300	3 (8.33)
-α^11.1kb^	NC_0000016.9:g. (220831_220860)_(231920_232003) del	2 (5.56)
IVS-I-117 (G>A)	HBA1:c.96-1G>A	1 (2.78)
CD 61 AAG>TAG	HBA2:c.184A>T	1 (2.78)
(αα)^YD^	NC_000016.9:144 094-197 947	1 (2.78)

Rearrangements and duplications of the α-globin gene cluster not included (described in text).

**TABLE 2:** Hematological data and α-lobin genotype of ten families.

Case	years	Hb (g/L)	MCV(fL)	MCH (pg)	Hb A2 (%)	Hb F (%)	Abnormal Hb	MMCA assay, MLPA or SMRT (for beta and alpha-globin gene cluster)
Family 1								
I-1(Father, Proband)	33	133	65.0	22.1	2.4			--SEA deletion; HBA2 gene converted to HBA1 gene
I-2(Mother)	37	123	67.2	20.8	2.4			--SEA deletion
II-1(Fetus)	13+6W							Normal
Family 2								
I-1(Father, Proband)	28	140.0	66.5	21.1	2.6			--SEA deletion
								the intron 2 of HBA2 converted to that of HBA1 gene
I-2(Mother)	26	113.0	68.1	20.6	2.3			--SEA deletion
I-3(Brother of father)	32	153.0	89.2	30.7	2.7			the intron 2 of HBA2 converted to that of HBA1 gene
II-1(Fetus)	12+2W							--SEA/αα
Family 3								
I-1(Father)	45	160.0	79.4	27.3	3.1			Duplications of α-globin gene (chr16:60,000-361,640)
I-2(Mother)	44	116.0	62.3	19.9	5.4			*HBB*: c.126_129delCTTT
II-1(Proband)	2	88.0	75.9	25.3	3.4	24.5		Duplications of α-globin gene ((chr16:60,000-361,640); HBB: c.126_129delCTTT
II-2(Fetus)	11+6W							HBB: c.126_129delCTTT
Family 4								
I-1(Father)	44	145	90.2	29.7	3.1			Duplications of α-globin gene
I-2(Mother)	44	116.0	62.3	19.9	5.4			HBB:c.216_217insA
II-1(Proband)	10	55.0	85.0	27.5	2.4	24.0		Duplications of α-globin gene
								HBB:c.216_217insA
II-2(Fetus)	12+3W							β^N^/β^N^
Family 5								
I-1(Father)	32	149.0	79.0	27.4	2.5			-α^4.2^/αα
I-2(Mother)	28	103.0	67.0	22.0	2.4			-α^11.1kb^/αα
II-1(Proband)	3	78.0	53.0	15.9	1.0		Hb H 8.10 Hb Bart’s 2.10	-α^11.1kb^/-α^4.2^
II-2(Fetus)	13+2W							αα/αα
Family 6								
I-1(Father)	28	144.0	68.2	21.1	2.3			--^SEA^/αα
I-2(Mother)	26	102.0	62.3	19.4	2.4			-α^11.1kb^/αα
II-1(Fetus)	24W							--^SEA^/-α^11.1kb^
Family 7								
I-1(Father)	29	131.0	65.5	20.1	2.4			--^SEA^/αα
I-2(Mother, Proband)	28	126.0	73.1	31.1	6.4	1.6		--^SEA^/αα chr11:5224211-523247 (GRch38/hg38)
II-1(Fetus)	13+6W							--^SEA^/αα
Family 8								
I-1(Father)	29	149.0	66.0	20.6	6.2	10.3		chromosome 11: 5224303-5227790 (GRCh38)
I-2(Mother)	29	132.0	76.1	24.9	5.2			β^−28 (A>C)^/β^N^
II-1(Proband)	1	53.0	67.7	27.1	5.4	81.2		chromosome 11: 5224303-5227790 (GRCh38) *HBB*:c.-78A>C
II-2(Fetus)	12+3W							β^−28 (A>C)^/β^N^
Family 9								
I-1(Father)	40	158.0	70.7	22.6	2.4	14.2		Chinese ^G^γ(^A^γδβ)^0^/β^N^
I-2(Mother)	36	109.0	72.8	22.8	5.2	1.5		HBA1、HBA2 deletion combined with HBB: c.126_129delCTTT
II-1(Fetus)	20+3W							Chinese ^G^γ(^A^γδβ)^0^ deletion combined with HBA1, HBA2 deletion
Family 10								
I-1(Father, Proband)	39	142.0	69.9	22.8	15.9			--^SEA^/αα
								Hb anti-Lepore Hong Kong
I-2(Mother)	36	127.0	64.1	22.0	2.4			--^SEA^/αα
II-1(Fetus)	13+6W							--^SEA^/αα

### 3.2 Ten family cases were further assessed

#### 3.2.1 Families 1 and 2: two rare micro gene conversions of the HBA2 gene

**FIGURE 2:**
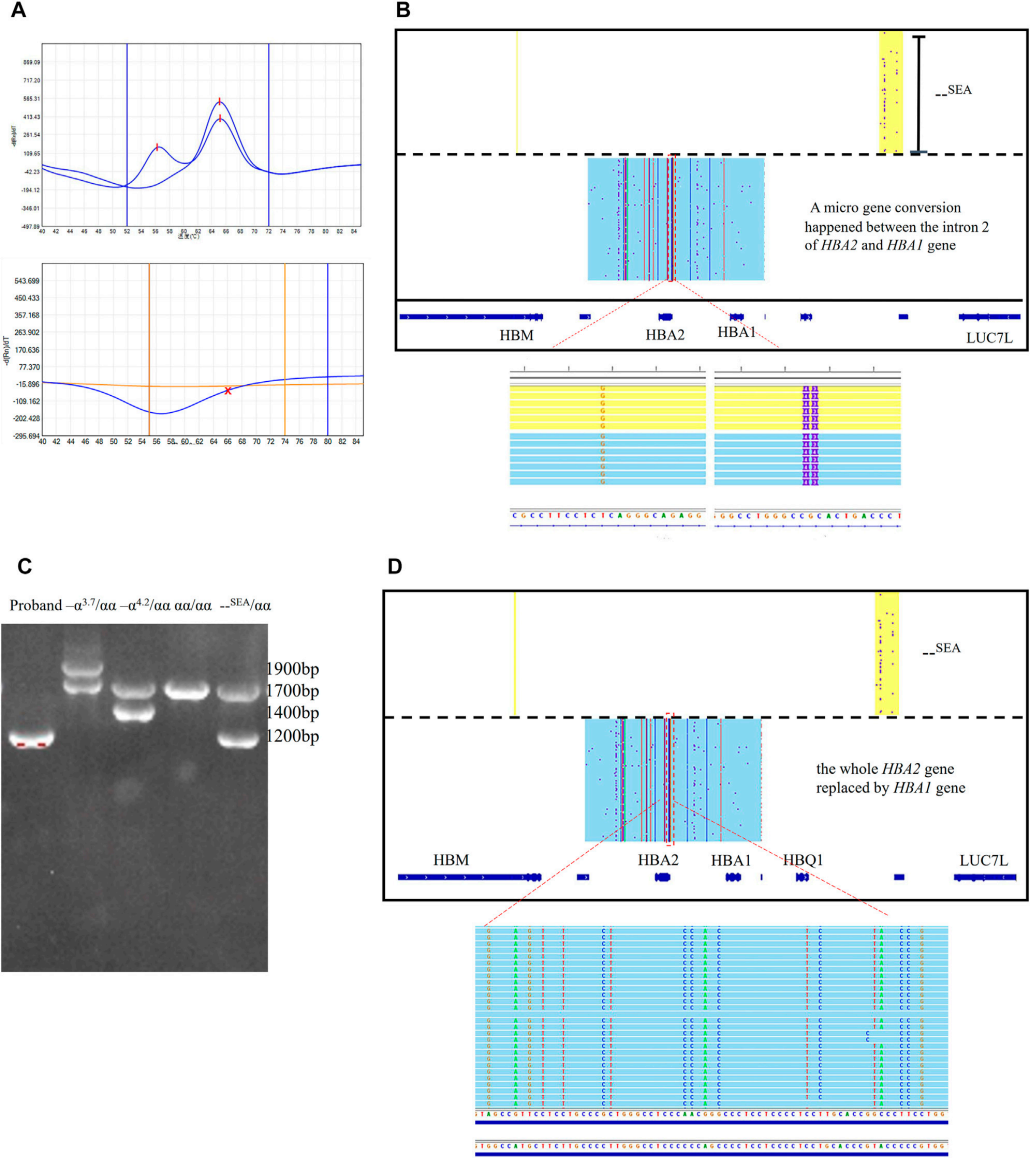
Rare micro gene conversions of HBA2 gene in family 1 and 2 **(A)** MMAC assay results for the probands in family 1. Two peaks were observed in the tunnel for--SEA deletion, indicating that he was the carrier with--^SEA^/αα. No peaks were observed in two tunnels for three common nondeletional α-thalassemia. **(B)** IGV plot showed one allele with–^SEA^ deletion in the yellow area and the other allele with micro gene conversions in α-globin genes in the blue area. The dashed red box highlighted was used to show that the intron2 in the *HBA2* gene was converted to the corresponding intron 2 sequence of the *HBA1* gene. **(C)** Gap-PCR result for the proband in family 2. Only the specific fragment for--SEA deletion was observed. The Gap-PCR results of three carriers with three common deletional α-thal and normal person were presented as references. **(D)** IGV plot showed one allele with–^SEA^ deletion in the yellow area and the dashed red box highlighted that the whole *HBA2* sequence was converted to HBA1.

Two couples suspected to be at high risk for severe α thalassemia were referred to our center. The wives were indicated to be--^SEA^/αα carriers through Gap-PCR. In both families, the result of the MMAC assay for deletional α thalassemia showed that the husbands were--^SEA^/αα carriers, but no amplification was observed using the RDB method and MMAC assay for three types of nondeletional α thalassemia. MLPA showed that both husbands carried a large deletion (Supplementary Figures S1A, B). The deletional range was similar with--SEA deletion, but the ratio of probes for intron 2 of the *HBA2* gene was near zero and that of the *HBA1* gene was one respectively. Genotypes of those two husbands were detected differently by using the SMRT sequencing method. The *HBA2* gene was fully converted to the *HBA1* gene in one person and a micro gene conversion was presented between the intron 2 of *HBA2* and *HBA1* genes in the other person (Figures 2A–D). We have also discovered the micro gene conversion in the spouse’s brother, who exhibits normal hematologic parameters and HbA2 levels. However, it is worth noting that this conversion did not occur in conjunction with the--SEA deletion observed in him.

#### 3.2.2 Families 3 and 4: two large duplications of the α-globin gene

**FIGURE 3:**
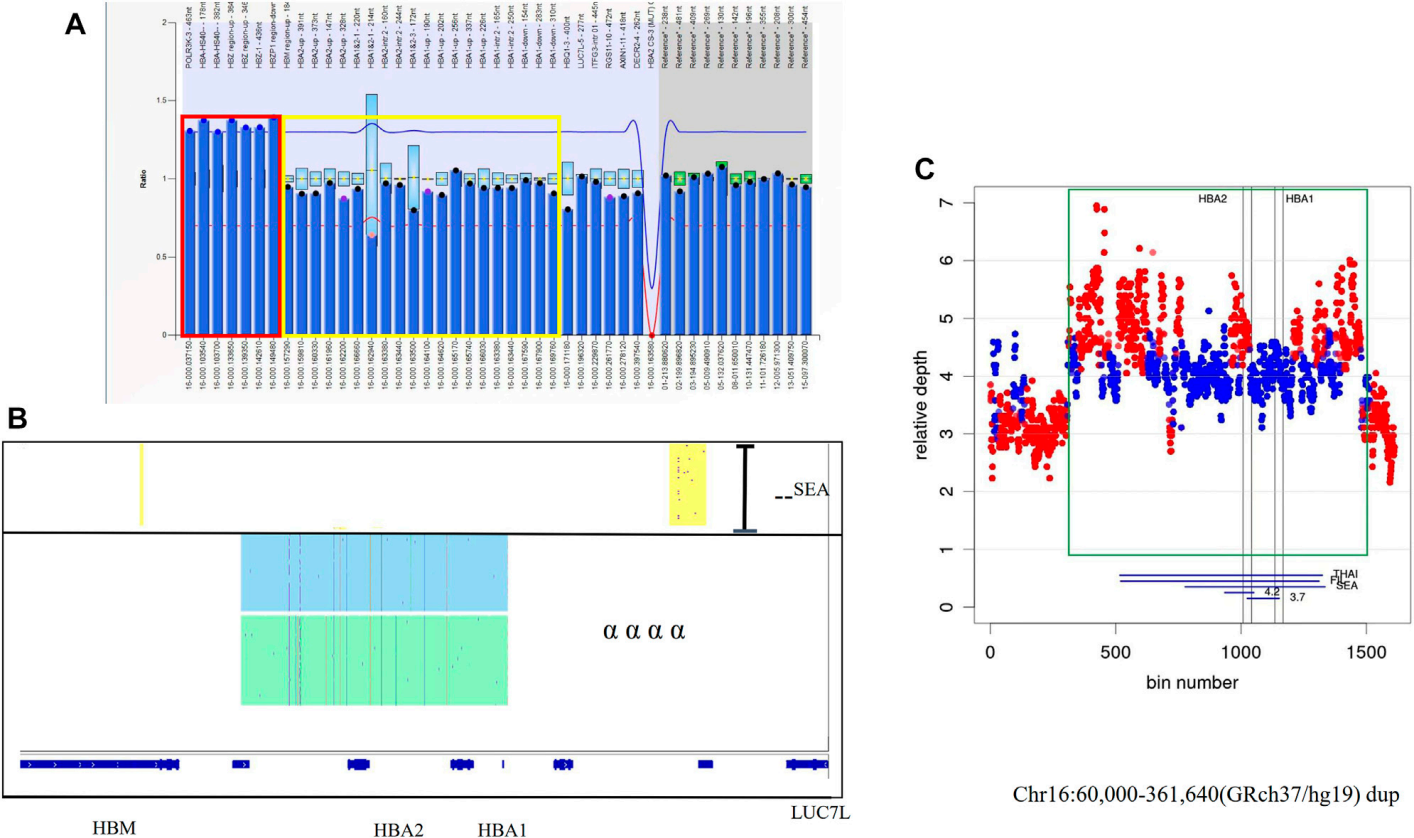
The large duplications of the α-globin gene in Family 3 **(A)** Duplication was observed in MLPA, the ratio signals of seven probes in the dashed red box were more than 1.25 which meant the existence of three copies. The proband had the normal ratio of the probes in the yellow box which was only 0.5 for the carrier with–SEA deletion. **(B)** IGV plot for the proband showed one allele with–^SEA^ deletion in the yellow area and two αα haplotypes with two different colored areas. **(C)** The coverage of WGS successfully defined a structural variation in chromosome 16:60,000-361,640 (GRCh37/hg19). showing The duplication region was shown in a green rectangle.

Two families came to our center for genetic counseling because they had given birth to a child with major thalassemia. Only one common mutation was found in the probands with β thalassemia major in those two families. The existence of duplications of the α-globin gene was found using MLPA in those two probands (Figure 3A). The results were also identified by SMRT sequencing, but the breakpoints could not be determined (Figure 3B). Based on Target Capture-Seq (TargetCap), the breakpoint was only determined in one of them (chr16:60,000-361,640) because the sequences were not captured in large repetitive sequences (Figure 3C).

#### 3.2.3 Families 5 and 6: two rare deletions of the α-globin gene cluster

Two pregnant women with abnormal reproductive histories came to our center for prenatal diagnosis. In one of the families, the proband was presented in a 3-year-old boy with moderate anemia. He received blood transfusions three times since he was born. Another proband clinically presented Hb Bart’s hydrops fetalis. MLPA showed a large deletion in the α-globin gene cluster, and its breakpoint had been identified by us using Target Capture-Seq previously (Jiang et al., 2021b). The genotypes of them were identified as -α^11.1kb^/-α^4.2^ and--^SEA^/-α^11.1kb^ using MLPA and Sanger sequencing, respectively (Supplementary Figures S3A–C). The prenatal diagnosis results showed that no deletions were found in fetus.

#### 3.2.4 Families 7 and 8: two large deletions of the β-globin gene cluster

**FIGURE 4:**
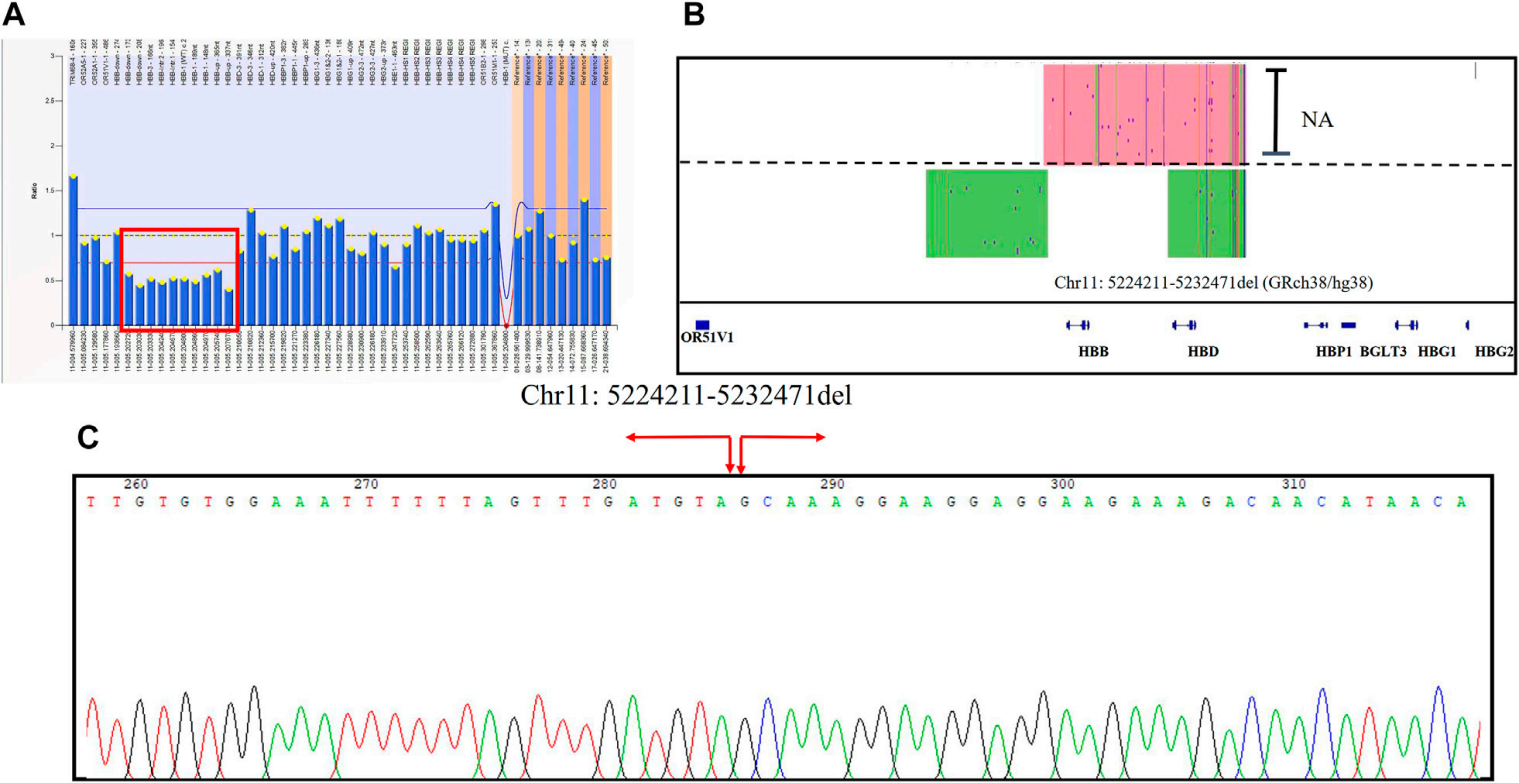
The large deletions of the β-globin gene cluster in Family 7 **(A)** Deletion was observed in MLPA, and the ratio signals of ten probes in the up-, down-stream, and intergenic regions of the *HBB* gene was 0.5 suggesting that the sample carried one copy of deletion (in red box). **(B)** IGV plot of the proband showed one allele with β globin gene in the red area and the other allele with the novel 8200 bp deletion in the green area. **(C)** Sanger sequencing was used to confirm the novel insertion. A red arrow was used to indicate the position of the novel deletion.

A 37-year-old pregnant woman with abnormal hematological parameters came to our center for genetic counseling. Hematological analysis results were abnormal, with a higher level of Hb A2 (6.5%). The result of the MMAC assay showed that she carries the thalassemia genotype--^SEA^/αα which is usually not the cause of high Hb A2 level. Sanger sequencing was used to exclude the existence of mutations in the *HBB* gene. MLPA was performed to detect novel deletions of β-globin clusters including the up-, down-stream, and intergenic regions of the *HBB* gene (Figure 4A). We first positioned the breakpoints at chromosome Chr11: chr11:5224211-523247 (GRch38/hg38) using SMRT sequencing analysis (Figure 4B), followed by identification using sanger sequencing through designed primers (Figure 4C). Considering the genotype of her husband is--^SEA^/αα, prenatal diagnosis was performed and excluded the existence of Hb Bart’s hydrops fetalis.

Another 27-year-old woman at 12 weeks gestation came to our center for prenatal diagnosis. She had a son with transfusion-independent anemia. The traditional method revealed that the proband was homozygous for *HBB*: c.-78 > G. The woman was heterozygous for the *HBB*:c.-78A>C β thalassemia variant, but no positive variant was detected in her husband using routine screening and Sanger sequencing. The husband suffered from microcytic hypochromic anemia with high levels of Hb A2 and HbF. The MLPA result indicated the presence of a heterozygous deletion in the male (Supplementary Figure S4A). Using the specific primer, we successfully determined the breakpoints at chromosome 11: 5224303-5227790 (GRCh38) also included up-, down-stream, and the whole *HBB* gene based on Sanger sequencing (Supplementary Figure S4B).

#### 3.2.5 Families 9 and 10: Two rare fusion genes in β globin cluster

**FIGURE 5:**
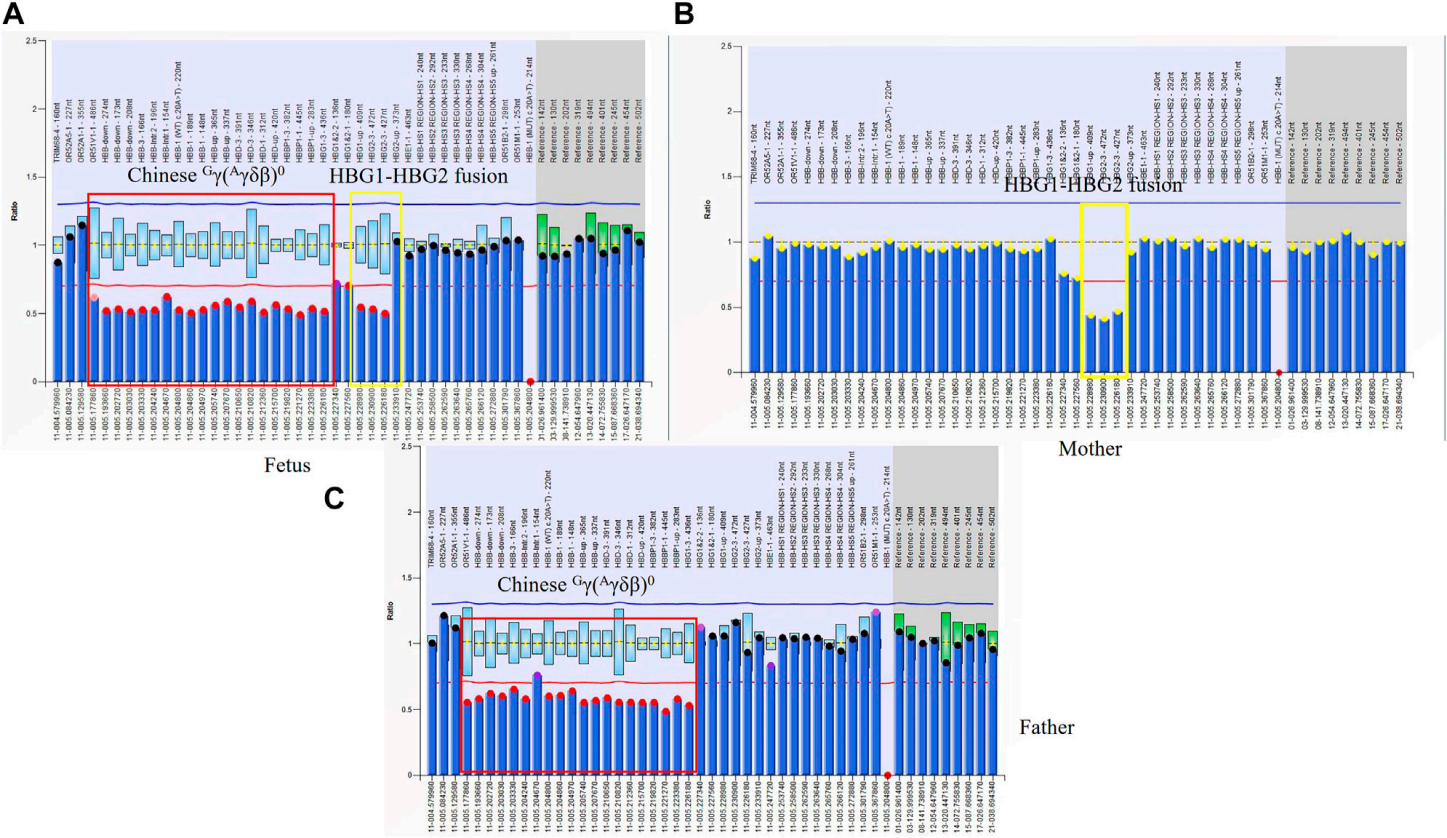
The rare fusion gene in β globin cluster in Family 9 **(A)** MLPA results for the fetus (II-1). The ratio signals of twenty probes in the regions including *HBB, HBD,* and part of the *HBG1* gene was about 0.5 suggesting that the sample carried Chinese ^G^γ+(^A^γδβ)^0^-thalassemia deletion (in red box). Interestingly, we also found another deletion including three probes in the region of the *HBG2* gene which we had described before. It was a 4.9 kb HBG1-HBG2 deletion in the β-globin gene cluster (hg38 chr11:5249345-5254268). A yellow box was used to indicate this deletion. There is about 520 bp between that two deletions. **(B)** In the family, the mother (I-2) had a ratio of 0.5 in those three probes suggesting that she carried one copy of rare deletion in the *HBG1-HBG2* area. **(C)** MLPA results for the proband (father). The ratio signals of twenty probes suggested he was the carrier with Chinese ^G^γ+(^A^γδβ)^0^-thalassemia deletion.

An 18-week pregnant woman suspected of being at risk of having children with beta-thalassemia major was seen at our center for prenatal diagnosis. Her previous genotype diagnosis was β^CD41-42(−CTTT)^/β^N^ combined with--SEA deletion and that of her husband was Chinese ^G^γ(^A^γδβ)^0^/β^N^. We performed gap-PCR for Chinese ^G^γ(^A^γδβ)^0^ thalassemia in them and the results showed that her husband and fetus were Chinese ^G^γ(^A^γδβ)^0^ thalassemia carriers. MMAC assay also identified the genotype of the wife and excluded codons 41/42 (–TTCT) (*HBB*: c.126_129delCTTT) mutation in the fetus. The MLPA results of the fetus and wife indicated the presence of a heterozygous deletion in the *HBG1-HBG2* region (Figures 5A–C). We obtained the fragments of *HBG1-HBG2* fusion using the special primers which we reported before (Jiang et al., 2020). The *HBG1-HBG2* fusion was first reported by our team and also described in detail by Qin Liu et al. (Jiang et al., 2020; Jiang et al., 2022). This is the first time to report the Chinese ^G^γ(^A^γδβ)^0^ thalassemia deletion combined with *HBG1-HBG2* fusion. The fusion caused a heterozygous deletion in *HBG1* and *HBG2* genes expressed during the fetal period. Interestingly, the deletion did not affect PCR amplification for detecting Chinese ^G^γ(^A^γδβ)^0^ thalassemia.

**FIGURE 6:**
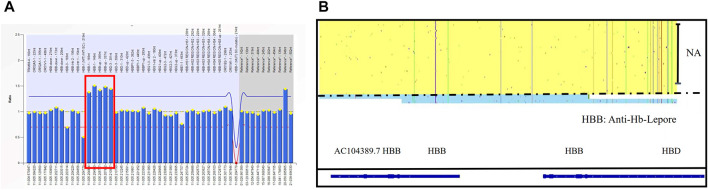
The rare fusion gene in β globin cluster in Family 10 **(A)** MLPA results for the proband (I-1). The ratio signals of five probes in the regions of the *HBB* gene and up the region of the *HBD* gene were more than 1.5 which suggested he carried three copies of rare duplication in the *HBB-HBD* area. **(B)** IGV plot of the proband showed one allele with β in the yellow area and the other allele with Anti-lepore in the blue area.

One at-risk couple was referred to our center due to the significantly high Hb A2 level of the husband. Both of them were diagnosed as--^SEA^/αα carriers, but the Hb A2 of the husband was 15.9%. Based on the result of MLPA and his phenotype, we speculated that he was a Hb anti-Lenore carrier (Figure 6A). Through SMRT sequencing technology, for the first time, we clearly obtained the complete sequence of 7,414 bp anti-Lenore Hong Kong (Figure 6B).

## 4 Discussion

Thalassemia is the most common birth defect in Guangdong province, China. It has been clarified to be very useful to perform prenatal diagnosis in couples who are carriers of the same type of thalassemia. Traditional methods for detecting population-specific targeted mutation are widely used in prenatal diagnosis. With the development of molecular biology methods, more and more at-risk couples with rare thalassemia variants have been offered prenatal diagnosis. Sanger sequencing and MLPA have been used in the prenatal diagnosis of rare thalassemia. The former is used for point mutation and the latter is applicable for CNV detection. Although targeted next-generation sequencing was reported to detect an additional 35 at-risk couples from 2522 subjects compared to traditional methods, it was rarely used in prenatal diagnosis of thalassemia due to high cost (Shang et al., 2017). A long-read sequencing-based approach was first used in prenatal diagnosis of thalassemia in 2022, which additionally identified α-globin triplicates in two fetuses with the heterozygous *HBB* c.316-197C>T variant (Luo et al., 2022a). The prenatal diagnosis rate of rare types of thalassemia was only 0.12% from 2011 to 2019 while it was 3.26% from 2020 to 2022, mainly because of the application of new genetic analysis technologies such as MLPA (Xu L. L. et al., 2020). The gene spectrum has also been changed especially for β-thalassemia. For example, Chinese ^G^γ(^A^γδβ)^0^ thalassemia/SEA-HPFH combined with point mutations in *the HBB* gene is the most common and rare point mutations in *the HBB* gene are a main cause for prenatal diagnosis in at-risk couples with a history of having children with severe β thalassemia syndrome (Xu L. L. et al., 2020). We found that about 1.13% of the couples were heterozygous for Chinese ^G^γ(^A^γδβ)^0^ thalassemia and SEA-HPFH in Guangzhou city. The most common characteristic of carriers with Chinese ^G^γ(^A^γδβ)^0^ thalassemia/SEA-HPFH is a high HbF level. Gap-PCR is a cost-effective method to detect those two types of thalassemia, especially in basic-level hospitals, thus we suggested traditional methods should cover those two genotypes (Jiang et al., 2020).

A combination of different methods was performed in this study. We additionally detected the complex genotypes of thalassemia which were different from previous results. The most representative one was a fetus with Chinese ^G^γ(^A^γδβ)^0^ deletion combined with *HBG2-HBG1* fusion using MLPA. His father was the carrier of Chinese ^G^γ(^A^γδβ)^0^ deletion while his mother was the--^SEA^/αα carrier coexisted with β^CD41-42(−CTTT)^/β^N^ based on the result from another hospital. The *HBG2-HBG1* fusion was not detected previously because the deletion (hg38, Chr11: 5226187-5231089) does not locate at the common Chinese ^G^γ(^A^γδβ)^0^ deletion locations (hg38 chr11:5169921-5248825). There is about 520 bp between those two deletions which does not affect primers for Chinese ^G^γ(^A^γδβ)^0^ deletion to bind. Using MLPA, we were able to simultaneously detect those two deletions. It is the first time that the *HBG2-HBG1* fusion was reported and found to cause the high HbF level by our team. We also found that the HbF was not presented in the proband with the same fusion described by Luo et al. (2022b). In this family, the HbF value was only 1.1% for the mother. The hematological phenotype of the woman and her 6-month-old baby described before was similar to that of ß-thalassemia minor genotypes, suggesting that *HBG1-HBG2* 4,924 bp deletion coexisted with *HBB* gene mutation does not cause the severe phenotype. The fetus was diagnosed with γ thalassemia because he had only one normal ^G^γ globin gene and an *HBG2-HBG1* fusion gene. The γ globin gene is mainly expressed during the fetal period, which includes the ^G^γ and ^A^γ globin genes. The ^G^γ^/A^γ ratio is about 7:3 in normal conditions, making the influence of the deletion in this fetus not severe (Nemati et al., 2010).

The importance of the combination of different methods is that we could identify different genotypes even if we obtain the same result based on one method. We have used MLPA to find a novel 8.2 kb heterozygous deletion in the β-globin gene cluster, but it was difficult to obtain accurate breakpoints. SMRT sequencing could accurately estimate the deletion range. The deletion only influenced the β-globin gene and its close upstream and downstream areas. Typically, there is a strong rivalry or competition for control between the promoter region of the β gene and the promoter regions of the γ and δ genes. The β gene promoter, responsible for the production of β globin chains, and the promoters of the γ and δ genes, responsible for the production of γ and δ globin chains, respectively, are vying for transcription factors to activate their respective genes. This competition determines the relative amounts of β, γ, and δ globin chains produced in a cell or tissue. The competition between these promoters can have important implications for the synthesis of hemoglobin, as different combinations of globin chains give rise to different types of hemoglobin. Thus, the deletion of the β-globin gene including the promoter may make the remaining γ- and δ-globin gene promoters react more readily with the enhancer elements and may result in higher levels of Hb A2. There were no significantly homologous sequences in the deleted region compared to the normal 5′and 3′sequences, suggesting that the deletion was caused by a non-homologous recombination event. There were AT-rich stretches around the 3′breakpoint, which may be significant in the recombination. A similar situation was observed in Taiwanese β-thalassemia and the case with a novel 7.2 kb deletion (Chr11:5222800-5230034, hg38) located in the *HBB* gene (Zhong et al., 2022). According to the phenotype of the proband and that of those two deletional β-thalassemia carriers, the deletion may cause β^0^ thalassemia. Interestingly, the result of MLPA for the novel 7.2 kb deletion was the same as the one we had described (Zhong et al., 2022). It has been shown that SMRT sequencing played an important role in thalassemia breakpoint detection. We also found another deletion which included up-, down-stream, and the whole *HBB* gene, which was recently reported in a Chinese family. The Hb A2 level and Hb F level were significantly high in patients with the two deletions, which was also observed in Taiwan deletion. Topfer et al. (2022) used CRISPR gene editing to delete elements within the *HBB* gene promoter and found that it could reduce *HBB* promoter activity, resulting in elevated fetal globin expression. However, whether the deletions in those regions could cause high HbA2 remained unclear. As rearrangement is very common in the α-globin gene cluster due to three homologous X-, Y-, and Z-box segments, it was difficult to discriminate the case with rare micro gene conversion of *HBA2* gene combined with--SEA deletion from the Hb Bart’s hydrops fetus using Gap-PCR. Gap-PCR was widely used in prenatal diagnosis and we previously reported a woman with this genotype using this method. The patient had classical mild α-thalassemia traits, suggesting the conversation would not influence phenotype. -α3.7 is the most common silent α-thalassemia and ααα^anti−4.2^ is the most common triplicated allele in China (Jiang et al., 2021a; Luo X. et al., 2021). In this study, we also obtained the same result of MLPA in those two rare micro gene conversions of the *HBA2* gene. Previous studies have shown that -α3.7 is the most common silent α-thalassemia and ααα^anti−4.2^ is the most common triplicated allele in China (Yin et al., 2014; Luo X. et al., 2021; Long and Liu, 2021). Based on our clinical observation and analytic results, we hypothesize that the genotype of one chromosome is -α3.7 and the other is ααα^anti−4.2^, and two Z homologous segments are unequally crossover and recombined to produce those two rare conversations (Supplementary Figure S2). Although the two rare micro gene conversions of the *HBA2* gene have been reported by our team, this is the first time to describe their phenotypes in family numbers. Our findings could give more information for prenatal diagnosis of thalassemia, especially rare types.

Duplications in the α globin gene combined with mutations in the β globin gene were another common reason for prenatal diagnosis of thalassemia (Shooter et al., 2015; Clark et al., 2018). MLPA, SMRT, and next-generation sequencing are useful methods for identifying large duplications in hemoglobinopathies (Jiang et al., 2022). It is difficult for MLPA to investigate chromosomal regions larger than 130 kb and determine the breakpoint. Compared to MLPA, SMRT, and next-generation sequencing can give precise information for the location of structural change in α and β globin genes simultaneously, except that they cannot capture the whole repetitive sequence (Ardui et al., 2018). Sequencing cost will also increase when more primer pairs are used in SMRT sequencing, which limits the detection range of the SMRT method to less than 10 kb (Luo S. et al., 2021). The situation also exists in targeted next-generation sequencing although the detection range is more than 100 kb. For the two rare cases reported in the study, more information about breakpoints could be obtained using targeted next-generation sequencing unless the breakpoint occurs in this sub-telomeric region of chromosome 16. The situation was also observed when we detected large deletions in α or β globin gene cluster.

--^THAI^ deletion was the most common deletion in prenatal diagnosis for rare α thalassemia in our study. MMAC assay includes this deletion but the result should be identified using another method. Another rare 11.2 kb deletion of the α-globin gene cluster was also common in prenatal diagnosis. It was first described in a case of Hb H disease by our team (Jiang et al., 2021b). Using Target Capture-Seq, the exact breakpoint location was detected and verified by Sanger sequencing using a special primer. The clinical phenotype of probands supplied by the 11.2 kb deletion could cause α^0^-thalassemia. Based on the two families, the phenotype of the rare 11.2 kb deletion carrier was similar to that of the–^SEA^ carrier. The result of follow-up for the proband with α^11.1kb^/-α^4.2^ supported that this rare deletion combined with other α^0^-thalassemia would not cause the severe clinical phenotype.

## 5 Conclusion

In conclusion, with the development of new sequencing technologies, more rare genotypes of thalassemia were detected, but this also provides a challenge for genetic counseling and prenatal diagnosis. In our study, we described the rare genotypes and clinical phenotypes in prenatal diagnosis. Chinese (^A^γδβ)^0^ and SEA-HPFH should be included in the detection range of conventional methods. It is important to identify the genotypes of parents before prenatal diagnosis, especially in cases with phenotypes not matching genotypes. MLPA for α globin cluster should be performed on cases with severe β− thalassemia who only carry a mutation in the *HBB* gene. Compared to other methods, SMRT sequencing has incomparable advantages to find complicated genotypes of thalassemia.

## Data Availability

The original contributions presented in the study are included in the article/Supplementary Material, further inquiries can be directed to the corresponding author.

## Supplementary material

SUPPLEMENTARY FIGURE S1 The results of MLPA for Probands in Family 1 and 2 **(A,B)** The same MLPA results for the probands in Family 1 and 2. The ratios for 24 probes were 0.5 except for two probes for the HBA2 gene and two probes for the HBA1 gene. Deletion in the region inside the red box suggested the existence of Asian (SEA)-type deletion according to the product description of SALSA® MLPA® probe mix P140-C1 HBA (MRC-Holland). The ratios for probes only detecting the HBA2 gene are nearly zero while the ratios for probes detecting the sequence in HBA1 are almost normal.

SUPPLEMENTARY FIGURE S2 The possible rearrangement mechanisms for two rare micro gene conversions of the HBA2 gene. We hypothesize that the genotype of one chromosome is -α3.7 and the genotype of the other chromosome is αααanti-4.2, and two Z homologous segments in those haplotypes are unequally crossover and recombined to produce those two rare conversations. For those two rare conversations, there was a slight difference in the unequal crossover region marked by broken lines with a different color. Exon 1 and intron 1 were the same for the HBA1 gene and the HBA2 gene.

SUPPLEMENTARY FIGURE S3 The results of MLPA and Sanger sequence for probands in Family 5 and 6 **(A)** The result of MLPA for proband in family 5. The ratios for probes marked by red arrow showed the South-East Asian (SEA)-type deletion according to Product Description SALSA® MLPA® probe mix P140-C1 HBA (MRC-Holland) while those by yellow arrow showed the rare -11.2 Kb deletion. **(B)** The results of MLPA for proband in family 6. The ratios for probes marked by the red arrow showed the 4.2 Kb deletion according to Product Description SALSA® MLPA® probe mix P140-C1 HBA (MRC-Holland) while those by the yellow arrow showed the rare −11.2 Kb deletion. **(C)** Sanger sequencing was used to confirm the rare 11.2 Kb deletion. The breakpoint for −11.2 Kb deletion was described.

SUPPLEMENTARY FIGURE S4 The results of MLPA and Sanger sequence for probands in Family 8 **(A)** The result of MLPA for proband in family 8. The ratios for 10 probes in the HBB gene marked by red arrow was about 0.5 suggesting that the sample carried the rare deletion. **(B)** Sanger sequencing was used to confirm the rare 3487 bp deletion. The breakpoint for 3487 bp deletion was described.
