# Supplementary figures and images for: Utilization of multiple genetic methods for prenatal diagnosis of rare thalassemia variants
Source: Front Genet. 2023 Jul 17;14:1208102. doi: 10.3389/fgene.2023.1208102 (PMC10387553; doi:10.3389/fgene.2023.1208102)

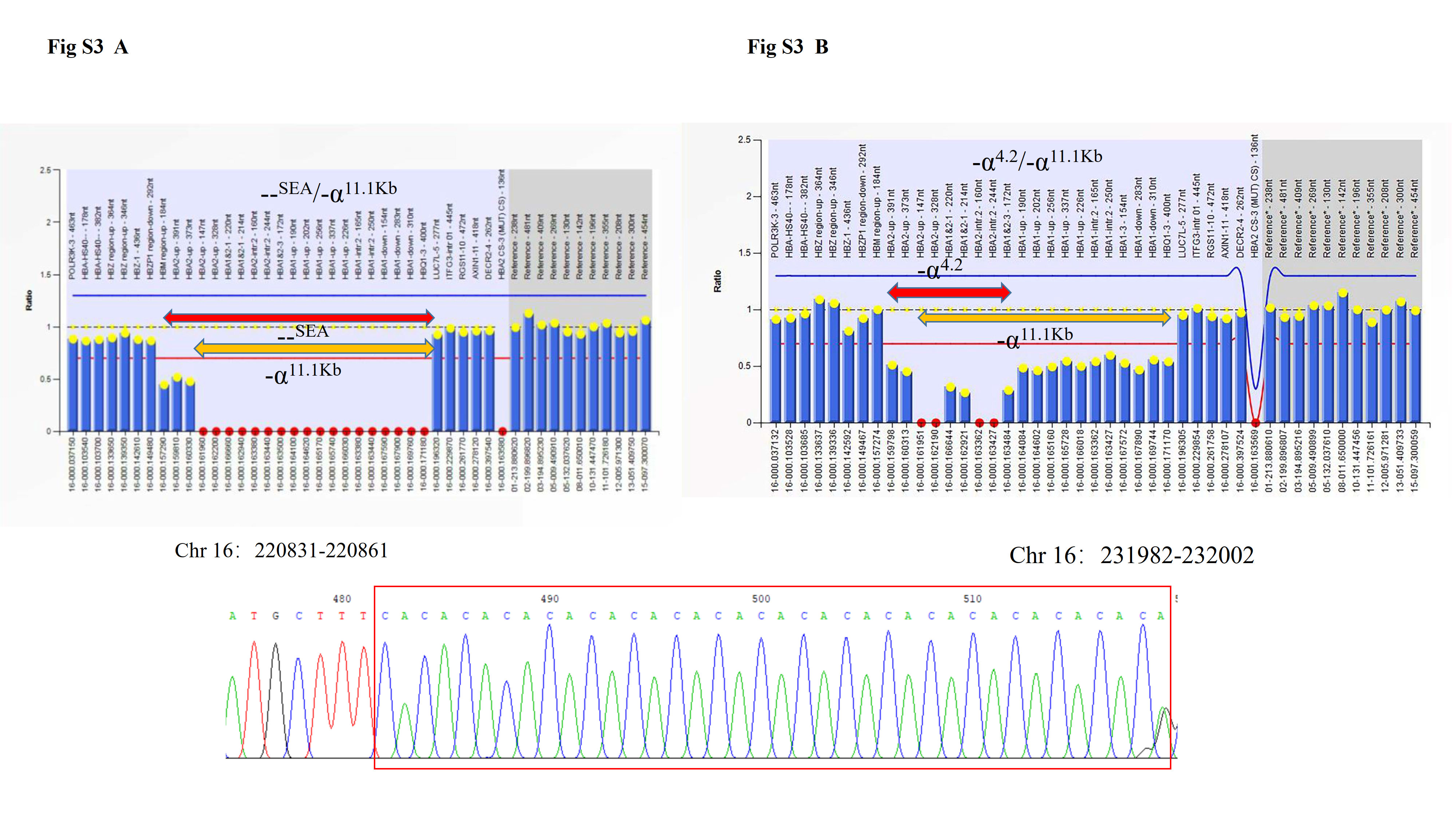

Supplement: Supplementary file 1 [file Image3.TIF]

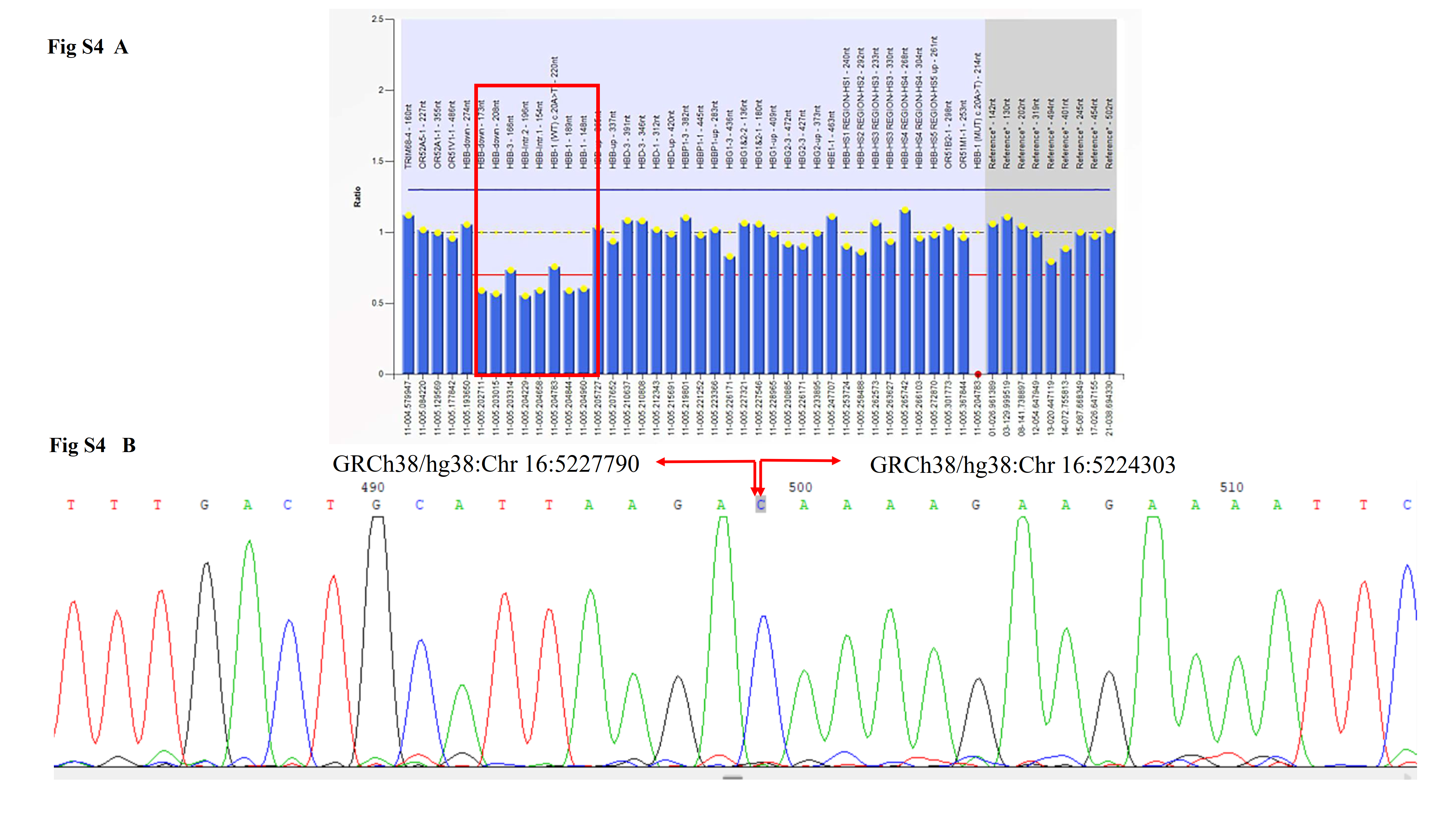

Supplement: Supplementary file 2 [file Image4.TIF]

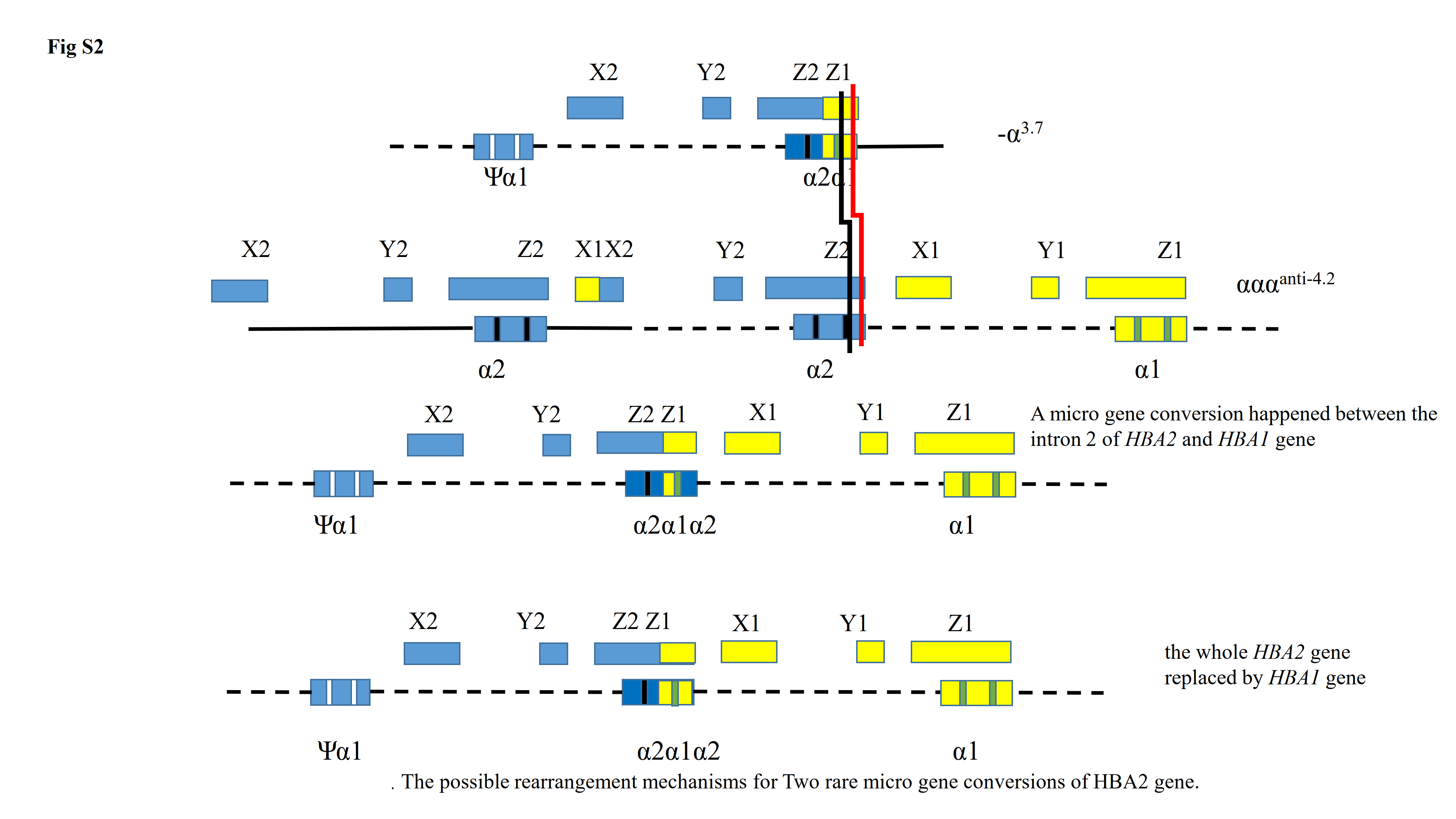

Supplement: Supplementary file 3 [file Image2.TIF]

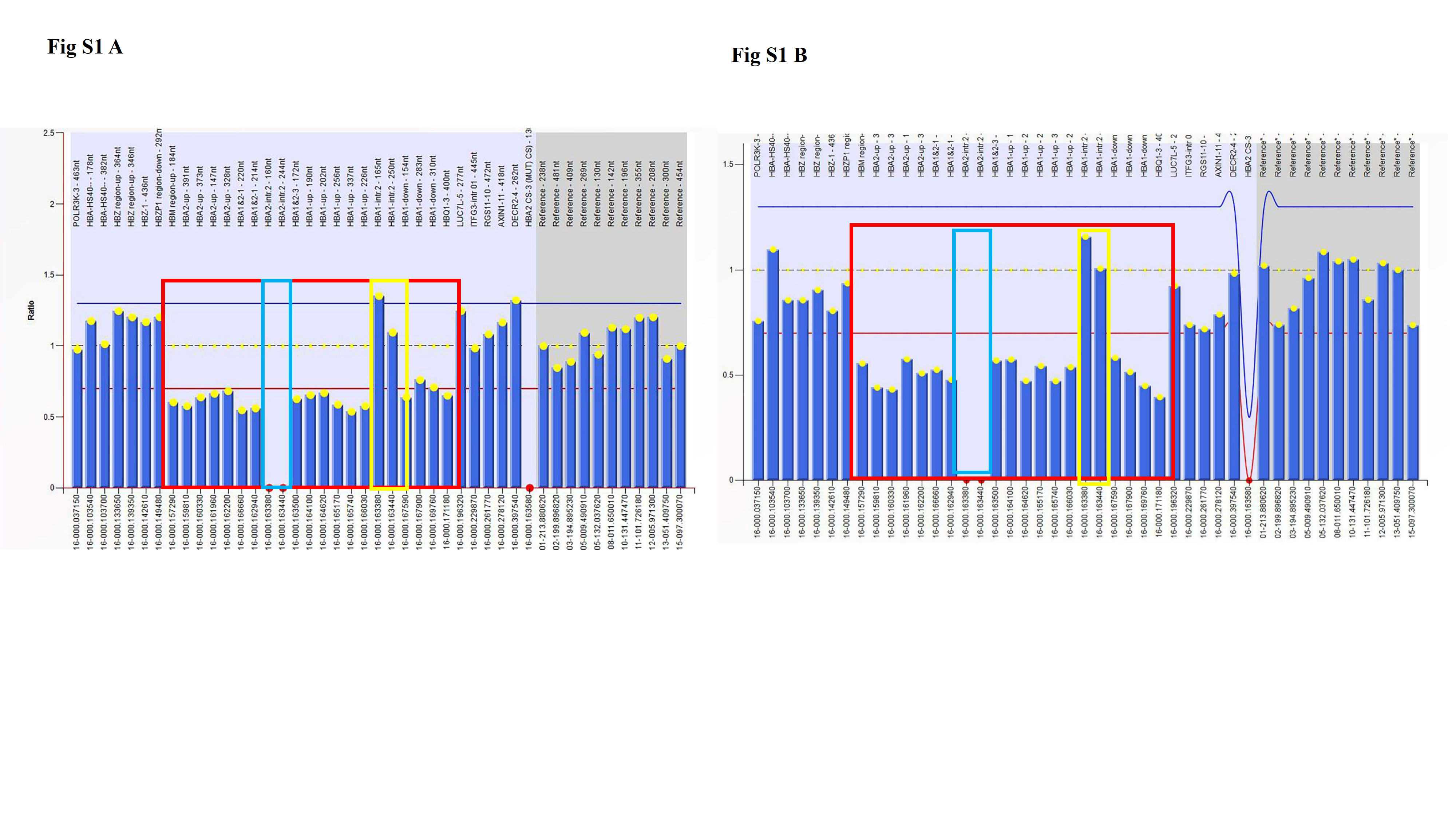

Supplement: Supplementary file 4 [file Image1.TIF]
